# Respiratory Strategies and Airway Management in Patients with Pulmonary Alveolar Proteinosis: A Review

**DOI:** 10.1155/2015/639543

**Published:** 2015-10-01

**Authors:** Tomas Vymazal, Martina Krecmerova

**Affiliations:** Department of Anaesthesiology and ICM, 2nd Faculty of Medicine, Charles University in Prague and Motol University Hospital, 150 00 Prague, Czech Republic

## Abstract

*Background*. Pulmonary alveolar proteinosis is a rare disorder characterized by a large accumulation of lipoproteinaceous material within the alveoli. This causes respiratory failure due to a restriction of gas exchange and changes in the ventilation/perfusion ratio. Treatment methods include noninvasive pharmacological approaches and invasive procedures, such as whole-lung lavage under general anesthesia. *Methods*. Based on the literature search using free-term key words, we have analyzed published articles concerning the perioperative management of adult and pediatric patients with pulmonary alveolar proteinosis. *Results and Discussion*. In total, 184 publications were analyzed. Only a few manuscripts were related to anesthetic, respiratory, and airway management in patients suffering from pulmonary alveolar proteinosis. Airway should be strictly separated using a double-lumen tube. Respiratory strategies involve the use of manual clapping, continuous positive airway pressure, high-frequency jet ventilation of the affected lung, and employment of venovenous extracorporeal membrane oxygenation in the most serious of cases. *Conclusion*. The goal of this review is to summarize the current published information about an anesthetic management strategy with a focus on airway management, ventilation, and oxygenation techniques in PAP patients.

## 1. Introduction

Pulmonary alveolar proteinosis (PAP) is a rare disorder first described by Rosen and colleagues in 1958 [1]. This disease is characterized by an accumulation of phospholipoproteinaceous material inside the alveoli due to a disruption in surfactant homeostasis. The prevalence of PAP is estimated to be around 4 cases per 1 million. Male gender, smokers, and adults between 30 and 50 years are most commonly affected. The symptoms due to a restriction in gas exchange and changes in the ventilation/perfusion ratio include shortness of breath (dyspnea), cough, and, in one-third of cases, also nail clubbing. The disease course may be complicated by opportunistic bacterial and fungal infections. There is a variable interindividual progression of this disease which can range from spontaneous recovery to terminal cardiorespiratory failure. Diagnosis is based on the findings from bronchoalveolar lavage (BAL), histology, and immunohistochemical tests and on the presence of granulocyte-macrophage colony stimulating factor (GM-CSF) antibodies and computed tomography (CT) or high resolution computed tomography (HRCT) results which typically shows areas of patchy ground-glass opacification and interlobular thickening which together produce the characteristic*‚* “crazy paving pattern” [2, 3]. PAP can also occur as an acquired disease (primary or idiopathic PAP), characterized by the production of GM-CSF antibodies, which is therefore autoimmune in origin. Congenital forms of PAP are less frequent and they are caused by a mutated gene responsible for surfactant production or by a mutated receptor for GM-CSF. Secondary forms of PAP are associated with other illnesses, mainly hematooncological diseases, exposure to inorganic materials (e.g., silicone), or caused by side-effects of medication, such as immunosuppressive drugs or amiodarone. Rarely, PAP can be a part of the acquired immune deficiency syndrome (AIDS) [4].

## 2. Therapeutic Approaches

There are two possible methods of the treatment of PAP depending on the etiology and severity of respiratory difficulties. Congenital, primary (idiopathic), and mild forms of PAP characterized by sufficient spontaneous breathing may be treated using a noninvasive, medical approach [4, 5]. The GM-CSF substitution is applied subcutaneously or inhalationally; other methods include biological treatment with monoclonal antibodies, plasmapheresis, or hyperbaric oxygen therapy [5]. Acquired or severe cases are associated with impairment of ventilation, dyspnea, development of severe hypoxemia, and hypercapnia and in these cases the only plausible treatment is lung lavage. In bronchoalveolar lung lavage, the lungs are flushed out with large quantities of saline. This procedure can be performed on individual lung segments, on lobes or on the whole lung with the option for longer periods between each lavage treatment. Whole-lung lavage (WLL) has been described as the most effective method of treatment [5–7]. The ultimate treatment for PAP is lung transplantation. Some published case reports suggest a successful treatment plan starting with WLL and continuing to GM-CSF application and/or other pharmacological treatment [5]. The first segmental washout was performed in 1963 by Ramirez et al. [8] and over the following five decades this procedure has been refined through the routine use of general anesthesia (GA) [4, 8], increasing lavage volumes and the use of warm saline and additional concomitant chest percussion and bilateral sequential WLL in the same treatment session [4]. In extreme situations, oxygenation can be supported using extracorporeal oxygenation (ECMO) [9]. In spite of the superiority of WLL as an interventional therapy for PAP, this procedure has not been yet standardized: it is not yet clear whether saline is superior to other solutions that are used, and each medical centre modifies its own practice [4].

## 3. Anesthetic, Respiratory, and Airway Management

Since 1965, WLL is routinely provided under general anesthesia (GA). GA presents a challenge due to the preexisting respiratory failure (with PaO_2_ being commonly lower than 6 kPa on room air) and the limited functional reserve of PAP patients [5, 6]. Anesthesiologists can take advantage of short acting anesthetics with the minimum of side-effects, analgesics, which are well tolerated by the patient, and reliable neuromuscular blockade recovery agents, which are all available for routine use in the operating theatre. Application of total intravenous anesthesia (TIVA) is recommended in these patients as it does not require the uptake of volatile anesthetics by the affected lungs and it enables the accurate conduction of GA during WLL [10]. Routine monitoring should include electrocardiography (ECG), invasive arterial blood pressure (IABP) measurement, and peripheral blood saturation (SpO_2_). Central venous line can be useful in blood gases analysis, intravascular volume assessment, and inotropic and vasoactive drugs administration. The CVP measures during pouring and draining the fluid in lateral position have limited value. Insertion of permanent urinary catheter with a temperature probe is recommended in procedures exceeding 4 hours. Point-of-care testing (POCT) of blood gases, sodium, potassium, chloride, magnesium, and osmolarity is recommended. Patients undergoing WLL can become hypothermic during these long procedures and therefore the appropriate management of body temperature is beneficial [11]. We also recommend transesophageal echocardiography (TEE) assessment prior to this procedure to evaluate cardiac function and during WLL for assessment of the lung tissue [12]. The manipulating TEE probe can move the DLT; therefore, immobilizing and fastening both the DLT and the probe are recommended. Based on our experience, PAP patients can suffer from pulmonary hypertension and symptoms of long-term right heart failure with an impact on left ventricular function. During WLL, an unpredictable volume of saline can be absorbed which may also overload the circulation.

### 3.1. Airway Management

Separation/isolation of lungs using double-lumen endotracheal tubes (DLT) for selective lavage of each lung and ventilation of the “dry” lung are necessary together with flexible bronchoscopy to confirm appropriate tube placement [11]. The use of double-lumen fiberoptic tubes (Vivasight ETView) have no superiority because bronchoscopic control of the lung tissue should be done by pneumologists prior to WLL. We have found that the optical lens can get smudgy immediately after the first wash cycle, thus becoming unusable for the remainder of the procedure.

Several case reports have described the use of serial lobar lavages with the use of a flexible bronchoscope together with endobronchial intubation of the healthy lung [13]. Davis et al. reported serial lavage in an awake patient under local anesthesia only [14].

Techniques for anesthetic management for WLL in children with PAP are determined by their age, weight, and severity of the disease. Pediatric airway management for WLL differs slightly from those techniques used in adults. Cuffed double-lumen tubes may be used for adolescents and school-age children. The smallest size of cuffed double-lumen tube which is available on the market has a diameter of 8.7 mm (26 F) [10]. This size may be used for pediatric patients older than 8 years of age [15]. Airway management techniques for younger children are usually very challenging. These include insertion of two separate narrow cuffed tracheal tubes inserted alongside into each bronchus or one of them endobronchially and the other one endotracheally [10, 16], insertion of a cuffed endobronchial tube into the healthy lung, with the lavage being performed with a flexible fibrescope inserted alongside into the other lung, and insertion of a standard cuffed tracheal tube with a cuffed bronchial blocker inserted into the affected main bronchus and the lavage performed via a gastric tube inserted alongside the bronchial blocker [17].

A special Marrano double-lumen tube which is available for smaller children cannot be used for WLL. This tube is uncuffed and therefore cannot protect the ventilated lung [10]. Neonates and infants undergoing WLL for pulmonary alveolar proteinosis are routinely managed with an endotracheal tube sitting above the carina and pulmonary artery flotation catheter inserted into the mainstem bronchus of the affected lung. The balloon of the catheter is then inflated and the lung is lavaged through it [18].

Some centers use the support of venovenous extracorporeal oxygenation (v-v ECMO) in the most difficult pediatric patients [19, 20]. Alternatively, WLL utilizing cardiopulmonary bypass (CPB) can be used in small children with severe form of PAP to support ventilation during lavage [17].

### 3.2. Respiration and Ventilation

Volume-controlled ventilation is preferred due to the higher rigidity and lower compliance of the affected lungs. Pressure-controlled modes would not guarantee the adequate ventilation. PAP patients suffer from hypercapnia and further elevation of CO_2_ partial pressure could affect microcirculation (the risk of brain oedema) and could provoke arrhythmias. The tidal volumes for each lung vary according to the condition of the pulmonary tissue, usually 200–350 mL at a rate of 12–16 breaths per minute in adults. In children, a sophisticated ventilatory regimen is not available due to the differences in age-based physiology and the degree to which the pulmonary tissue is affected [10, 12, 17]. The adjustment of end-tidal PCO_2_ or better PaCO_2_ (due to a restriction of gas exchange and changes in the ventilation/perfusion ratio in PAP) is recommended [21, 22]. Positive end-expiratory pressure is usually set between 5 and 7 cmH_2_O. The FiO_2_ in the inspired gas mixture is adjusted according to PaO_2_ values, with fractions higher than 0.6 often required [6, 12]. In one case report, the authors applied continuous positive airway pressure (CPAP) ventilation into the washed lung, aiming to improve oxygenation [23]. Manual clapping performed by a respiratory physiotherapist during WLL can help to clear out the protein-rich alveolar material from the affected lung tissue [24]. This technique has been found to be superior to mechanical chest percussion or to WLL without any chest percussion. Several case reports have described the successful perioperative use of a novel system for clearance of the airway secretions in patients with PAP. The vest employs complex technology in which increased airflow velocities are generated, thus creating shear forces similar to spontaneous coughing while simultaneously reducing viscosity of secretions including the lavaged material [25, 26]. Oxygenation in the most severely affected adult and pediatric patients may be achieved with the aid of extracorporeal oxygenation techniques, v-v ECMO, or even full cardiopulmonary bypass (CPB), regardless of the risk of significant morbidities [10, 17].

Other methods supporting ventilation and oxygenation during this procedure have been tested only in experiments. Partial liquid ventilation (PLV) using perflubron improved oxygenation only temporarily and had no positive effect on protein removal in an infant with PAP scheduled for WLL [27]. Hyperoxygenated saline solution was found to improve oxygenation in five patients undergoing WLL for PAP in comparison with normal saline [28].

The duration of WLL is usually several hours and the accumulated phospholipoproteinaceous material is washed out of the lungs with 0.5–2.0 L of body temperature warmed saline in aliquots for 10–30 wash cycles per each lung. The total volume of saline can reach 30 liters [11, 29]. The patient must be positioned step by step into the right decubitus position and subsequently onto the left side according the protocol recommended by the American College of Chest Physicians in 2009 [11]. The aliquots of saline are subsequently loaded into the inferiorly placed lung following 4–6 minutes of percussion postural massage of the chest wall with the aim of evacuating the maximal amount of accumulated material. Afterwards, the effluent is drained with the help of gravity by placing the patient into the Trendelenburg position (“head down”). This procedure has to be repeated until the rinsed fluid is clear. The same algorithm is applied to the other lung [11, 12]. The volume of solution is essential. If the volume of solution was high enough, pulmonary blood flow shifts to the ventilated lung, and as a result the oxygenation improved. This shift of blood flow became smaller during the drainage phase, and oxygenation became worse. The nature is the hypoxemic pulmonary vasoconstriction effect and the bypassing of the affected lung. The adequate volume also facilitates effectiveness of the WLL. Validated data about the absorbed volume of saline is missing in the published cases. Extensive suction of the residual solution is required for the better oxygenation after procedure. The amount of chloride absorbed has not also been evaluated in any published report. We noted the development of significant facial soft tissue oedema during WLL as a possible result of absorption of electrolytes into the third space. The WLL procedure can be accompanied by derangements of acid-base

and electrolyte balances or hypervolemia. The type of crystalloid to be used in this procedure has not yet been standardized. Therefore, hourly electrolyte assessment can be recommended in WLL cases with extensive NaCl lavage considerations. Derangement of the electrolyte homeostasis causing cardiac arrhythmias might be avoided using Ringer's solution [30].

### 3.3. Management of Severe PAP

Whole-lung lavage can also be performed with ECMO or CPB support in adult and pediatric patients. The decision depends on the severity of clinical symptoms [31]. Most commonly, the cannulae used in v-v ECMO are placed such that the infusion cannula is inserted in the right internal jugular vein and the drainage cannula in the right femoral vein [32]. In the case of heart failure, it is possible to choose venoarterial ECMO [33]. Cannulae for venoarterial ECMO are then placed using either a percutaneous or surgical technique. In the literature, there is a case of WLL and lung resection being performed simultaneously, although the operation was complicated by cardiac arrest due to hypoxia. After successful CPR, the procedure was continued with the support of v-v ECMO [34]. Adequate oxygenation with the support of ECMO allowed the completion of the procedure and prevented cardiac arrhythmias and hemodynamic instability. It also avoided the complications that may occur in the interval from the end of procedure to the restoration of the lung tissue with traditional techniques of mechanical ventilation [9, 31]. v-v ECMO cannulation is an invasive technique with many possible complications such as bleeding, arterial puncture, vessel rupture, or development of pseudoaneurysms. WLL may be also complicated by pneumothorax. However, it remains the only possible technique that prevents serious hypoxia during WLL. Even when taking into account all of the risks connected with WLL and v-v ECMO support, this technique is very effective [32, 35].

## 4. Conclusions

This review has aimed to present an anesthetic management strategy with a focus on airway management, ventilation, and oxygenation techniques for easier perioperative decision-making in patients scheduled for whole-lung lavage under general anesthesia due to pulmonary alveolar proteinosis. Further prospective studies or larger cohort studies are needed to obtain more robust data from which it is hoped that further guidelines could arise.

## Abbreviations

AIDS: Acquired immunodeficiency syndrome
BAL: Bronchoalveolar lavage
CPAP: Continuous positive airway pressure
CT: Computed tomography
ECMO: Extracorporeal membrane oxygenation
GA: General anesthesia
GM-CSF: Granulocyte-macrophage colony stimulating factor
HRCT: High resolution computed tomography
PAP: Pulmonary alveolar proteinosis
PEEP: Positive end-expiratory pressure
POCT: Point-of-care testing
TEE: Transesophageal echocardiography
TIVA: Total intravenous anesthesia
VCV: Volume-controlled ventilation
v-v ECMO: Venovenous extracorporeal membrane oxygenation
WLL: Whole-lung lavage.
